# Beyond flow: a ventricular-load framework for VA-ECMO in cardiogenic shock

**DOI:** 10.3389/fmed.2026.1937424

**Published:** 2026-10-23

**Authors:** Rachid Attou, Sébastien Redant, Ikram Boutrika, Sohaïb Mansour, Mohamed El Mallouli, Leticia Al Kai, Ayoub Jaafari

**Affiliations:** 1 Department of Intensive Care, CHU Brugmann, Université Libre de Bruxelles, Brussels, Belgium; 2 Department of Cardiology, CHU Brugmann, Université Libre de Bruxelles, Brussels, Belgium

**Keywords:** cardiogenic shock, left ventricular unloading, mechanical circulatory support, myocardial recovery, VA-ECMO

## Abstract

Cardiogenic shock still carries a 30-day mortality of approximately 40%–50% despite reperfusion, vasoactive support and modern intensive care. Veno-arterial extracorporeal membrane oxygenation (VA-ECMO) can rapidly restore systemic perfusion, but recent randomised evidence has reframed its role. In infarct-related shock, randomised trials have not shown a clear survival benefit from routine early VA-ECMO; ECLS-SHOCK and pooled analyses also found more bleeding and vascular complications. In the same period, DanGer Shock showed that a primary microaxial-pump (Impella CP) strategy improved survival in a carefully selected population with ST-elevation myocardial infarction complicated by cardiogenic shock, although it was tested against standard care rather than head-to-head against VA-ECMO and does not isolate unloading as the causal mechanism. Read together, these data motivate the central argument of this review: peripheral VA-ECMO is usefully managed not only as a problem of circulatory flow, but also as a dynamic problem of ventricular loading. We advance this as an organising framework rather than as a demonstrated mechanism, since differences in patient selection between these trials offer a competing and equally tenable explanation. Retrograde aortic return increases effective LV afterload, while venous drainage reduces preload; the clinical result depends on the interaction between circuit flow, native contractility and biventricular loading conditions. This narrative, non-systematic review synthesises contemporary trial evidence, the physiology and treatment of LV loading, and approaches to serial assessment of the supported heart, from haemodynamics and bedside echocardiography to emerging deformation and advanced imaging tools. VA-ECMO should therefore be viewed as a time-limited bridge whose net benefit depends on reversible disease, early recognition of clinically relevant ventricular overload and repeated assessment of recovery, escalation options and futility.

## Introduction

Cardiogenic shock is defined by tissue hypoperfusion from primary cardiac failure despite adequate filling, and its mortality remains 40%–50% despite reperfusion, vasoactive support and modern intensive care (1, 2). Veno-arterial extracorporeal membrane oxygenation (VA-ECMO) provides rapid temporary circulatory support and can maintain systemic perfusion while myocardial recovery, transition to durable mechanical support, transplantation or limitation of therapy is considered (3). Its use expanded rapidly before robust randomised evidence became available (4). When randomised evidence emerged in 2023–2024, the results were sobering. In infarct-related shock, routine early VA-ECMO did not improve survival in the randomised trials; ECLS-SHOCK and pooled analyses also reported more bleeding and vascular complications (5–9). In contrast, DanGer Shock showed that a primary microaxial-pump (Impella CP) strategy improved survival in a carefully selected population with ST-elevation myocardial infarction complicated by cardiogenic shock; this was a trial of microaxial-pump support against standard care, not a head-to-head comparison with VA-ECMO (10).

Read together, these trials shift the question from “does VA-ECMO work?” to “which form of support best matches the patient's physiology, and how should its loading consequences be managed?” Peripheral VA-ECMO restores systemic flow but can increase effective LV afterload through retrograde aortic return. The neutral results of routine VA-ECMO and the benefit observed with a primary microaxial-pump strategy in DanGer Shock raise the hypothesis that ventricular loading conditions are clinically important, but they do not establish unloading as the causal explanation for the divergent trial results. We therefore use ventricular load as a physiological framework for bedside decision-making, reorienting management from “how much support?” toward “how is this support altering biventricular load, and is the myocardium recovering?”

This reorientation highlights an incompletely standardised area of practice. Guidelines and reviews agree that VA-ECMO should be time-limited and goal-oriented, with repeated reassessment of recovery, bridge options and futility, but operational criteria remain heterogeneous. In practice, serial basic echocardiography and biochemical trajectories are often central to these decisions. Advanced echocardiographic deformation imaging and molecular or nuclear imaging, which are used in other settings to characterise myocardial function, viability and prognosis, remain only sparsely studied during mechanical support. This review is organised around that gap: after summarising pathophysiology, indications and contemporary randomised evidence, we focus on ventricular loading and unloading and then examine how the supported heart can be assessed serially, from haemodynamics and bedside echocardiography to emerging imaging approaches.

## Methods

This is a narrative (non-systematic) review. We searched PubMed/MEDLINE, Embase and the Cochrane Library from inception through August 2026, combining the terms “cardiogenic shock”, “extracorporeal membrane oxygenation”, “VA-ECMO”, “left ventricular unloading”, “Impella”, “weaning”, “myocardial recovery”, “pulmonary artery catheter”, “lung ultrasound”, “deformation imaging”, “computational fluid dynamics”, “reduced-order modelling” (including “reduced-order model”, “lumped-parameter” and “one-dimensional” models) and “mock circulatory loop”, and hand-searched the reference lists of key articles and relevant society guidelines. We prioritised randomised controlled trials, individual-patient-data and other meta-analyses, and current guidelines or consensus statements, drawing on selected observational cohorts and physiological studies where randomised data were lacking. Engineering and simulation studies (computational fluid dynamics, reduced-order circulation models and *in vitro* mock circulatory loops) were included when they addressed aortic flow mixing, ventricular loading or oxygen delivery during VA-ECMO. English-language adult studies were prioritised. Given the narrative design, articles were selected for relevance and methodological quality rather than by a prespecified systematic protocol, and no formal risk-of-bias scoring was undertaken.

## Pathophysiology and circuit principles

A VA-ECMO circuit drains venous blood, oxygenates it and removes carbon dioxide across a membrane oxygenator before returning it to the arterial system (3, 11). Delivered flow depends on venous preload, cannula and circuit resistance, and centrifugal pump speed. In the standard peripheral (femoro-femoral) configuration, deoxygenated blood is drained from the right atrium via the femoral vein and reinjected into a femoral artery, generating retrograde flow in the descending aorta (12). When native LV ejection is present, antegrade native flow and retrograde extracorporeal flow are competitive flows that meet at a mixing point whose location depends primarily on their relative pressure-flow contributions and the arterial return site (13) (Figure 1). This creates dual circulation, with a native-lung zone proximal to the mixing point and a membrane-lung zone distal to it; regional differences in oxygen and carbon dioxide content may therefore occur when gas exchange differs between the native and membrane lungs (13).

**Figure 1:**
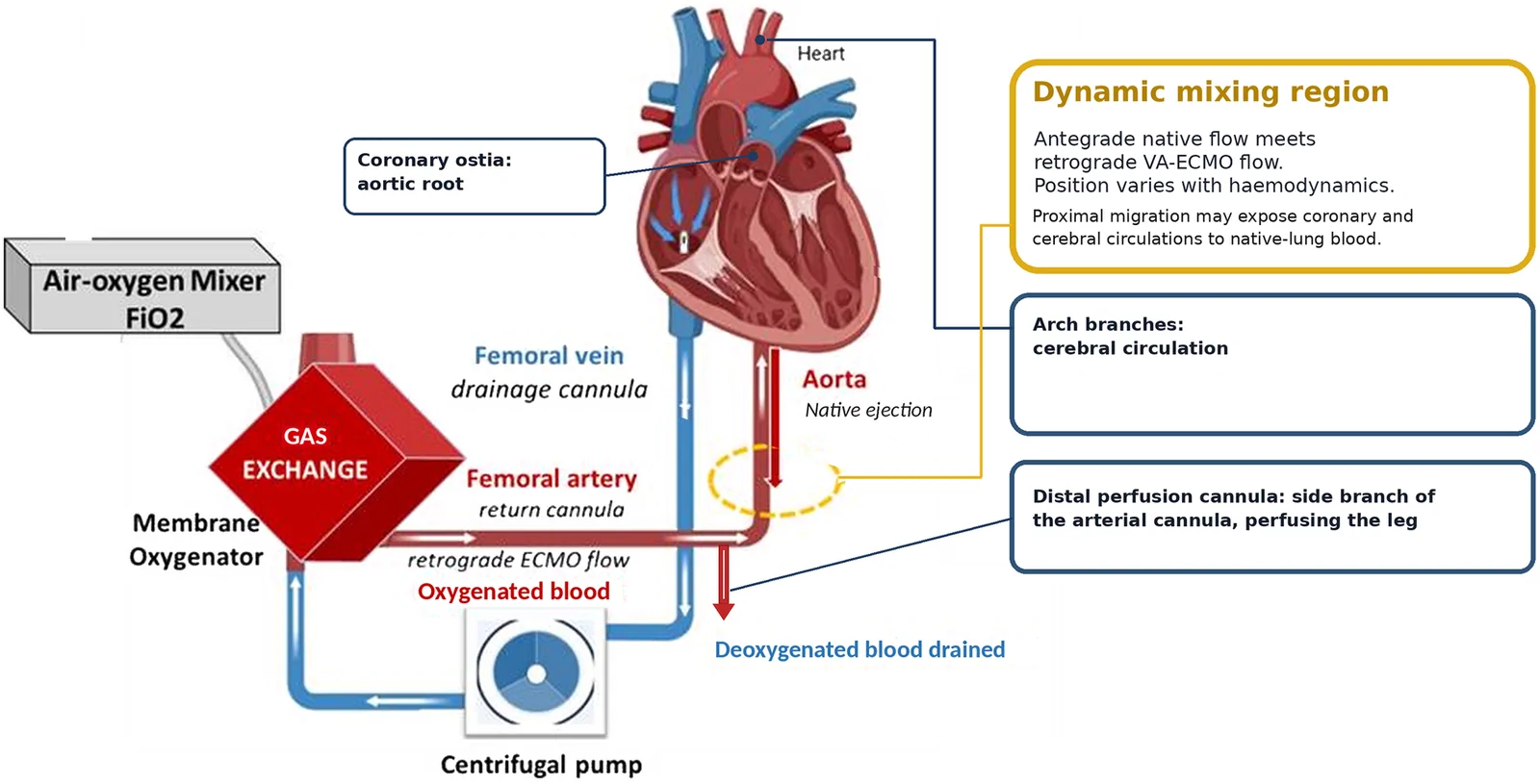
Peripheral VA-ECMO circuit and dynamic aortic mixing. Venous blood is drained to the pump-oxygenator and returned through the arterial cannula as retrograde oxygenated flow. Native LV ejection generates antegrade aortic flow. Their interaction creates a dynamic mixing region rather than a fixed anatomical “watershed”; its position depends on ECMO flow, native cardiac output, aortic pressure/impedance, cannula position and vascular anatomy. Proximal migration of the mixing region in the presence of impaired pulmonary gas exchange may expose the coronary and cerebral circulations to less oxygenated native blood; the coronary ostia at the aortic root and the arch branches supplying the cerebral circulation are labelled separately. A distal perfusion cannula maintains perfusion of the cannulated limb (13–17).

## Left ventricular overload and unloading

VA-ECMO alters LV loading on both sides of the pressure-volume loop. Venous drainage lowers RV preload and may reduce pulmonary venous return and LV preload, whereas retrograde arterial return raises effective LV afterload. The net effect cannot be predicted from pump flow alone: LV volume, filling pressure, stroke volume and wall stress also depend on drainage effectiveness, intravascular volume, vascular tone, pulmonary blood flow, biventricular interaction and native contractile reserve. Thus, clinically important LV distension can occur despite substantial right-sided decompression, particularly when native contractility is poor and the aortic valve does not open. Residual bronchial and Thebesian return, pulmonary venous return and aortic regurgitation may continue to fill the LV (13, 18).

Peripheral VA-ECMO can increase effective LV afterload through retrograde aortic return, particularly as arterial pressure and extracorporeal flow increase. When the ventricle cannot open the aortic valve against this load, intracavitary pressure and wall stress may rise, the valve may remain closed, and LV distension with elevated end-diastolic pressure and pulmonary congestion may increase myocardial oxygen demand and impair subendocardial perfusion, potentially hindering myocardial recovery (18, 19) (Figure 2A). Early recognition rests on echocardiography, looking for a dilating, poorly ejecting ventricle, spontaneous echo contrast, persistent aortic valve closure and worsening pulmonary oedema, complemented by lung ultrasound (bilateral B-line pattern).

**Figure 2:**
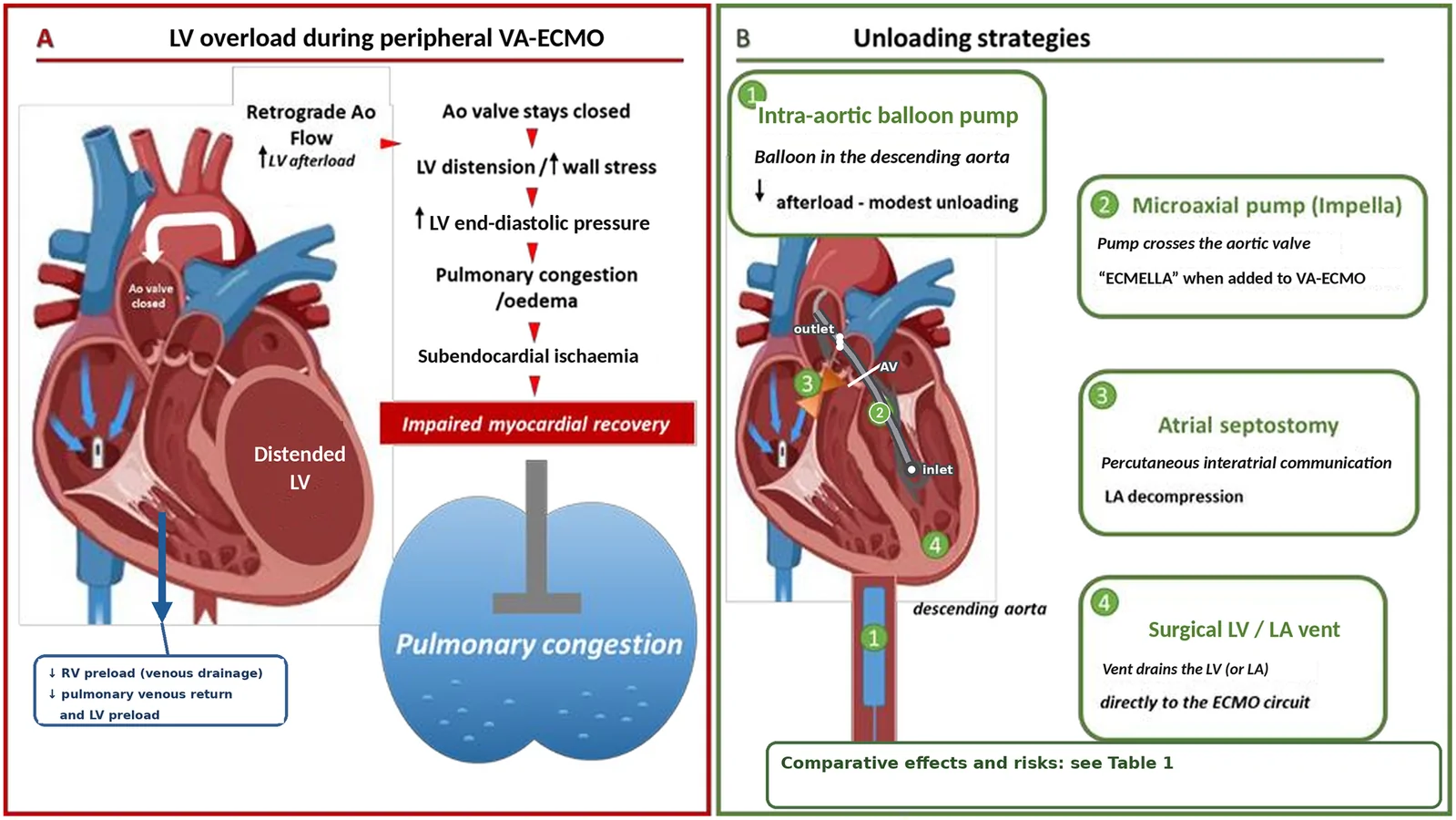
Left ventricular overload during peripheral VA-ECMO **(A)** and unloading strategies **(B)**. **(A)** Retrograde aortic flow raises left ventricular afterload; when the ventricle can no longer open the aortic valve it distends, end-diastolic pressure and wall stress rise, and pulmonary congestion with subendocardial ischaemia may impair recovery. The net loading state, however, is the resultant of this afterload rise and the preload reduction produced by right atrial drainage (shown in panel A), and distension therefore occurs chiefly when native contractility is low, pump flow high, or ventricular filling continues through aortic regurgitation and bronchial and Thebesian return. **(B)** Four mechanical approaches can unload the ventricle: the intra-aortic balloon pump, a microaxial pump (Impella; “ECMELLA” when combined with VA-ECMO; shown across the aortic valve), atrial septostomy or transseptal left atrial venting, and surgical venting. Their comparative haemodynamic effects, bleeding and access profiles and resource requirements are summarised in Table 1.

**Table 1:** Comparative overview of principal left-sided unloading approaches during VA-ECMO. The magnitude of decompression is strongly configuration- and flow-dependent. Evidence comparing modalities is predominantly observational; no adequately powered randomised head-to-head trial has established a superior strategy (18–22, 28).

Modality	Haemodynamic effect on LV load	Typical vascular/bleeding profile	Anticoagulation/device-related considerations	Cost/accessibility
Intra-aortic balloon pump (IABP)	Indirect, relatively modest LV unloading through systolic afterload reduction with diastolic coronary augmentation; reduction in hydrostatic pulmonary oedema has been reported during peripheral VA-ECMO (29)	Smaller arterial access than Impella; limb ischaemia and bleeding remain possible	Usually compatible with the overall ECMO anticoagulation strategy; no purge system	Widely available in many centres; generally lower device cost than microaxial pumps
Microaxial pump (Impella; “ECMELLA/ECPELLA”)	Active, direct LV decompression; magnitude depends on pump position, pump flow, native loading conditions and interaction with ECMO flow	Second large-bore arterial access; bleeding and haemolysis are important concerns, with vascular complications also possible	Requires integration of ECMO anticoagulation with device-specific purge/anticoagulation management; bleeding risk reflects both pharmacological and access/device factors	Higher device/resource cost; requires specific implantation and monitoring expertise
Transseptal left atrial decompression	Direct reduction of left atrial pressure and LV preload via drainage/shunting; magnitude depends on cannula/shunt characteristics and circuit configuration	Avoids a second arterial large-bore access; procedural risks include septal puncture complications, bleeding and residual shunt	Usually no separate long-term anticoagulation target, but procedural bleeding/thrombotic risk and overall ECMO anticoagulation still require individual assessment	Requires transseptal and echocardiographic expertise; availability is centre-dependent
Surgical LV vent	Direct LV or LA decompression with potentially substantial unloading; magnitude depends on vent location, size and drainage flow	Most invasive option; surgical bleeding and chest-access/re-exploration risks	Depends on the concurrent surgical anticoagulation strategy	Requires cardiac surgical capability; mainly available in surgical/central-ECMO settings

Unloading may be achieved with an intra-aortic balloon pump (IABP), a microaxial pump such as Impella (the “ECMELLA” or “ECPELLA” combination), transseptal left atrial decompression or a surgical vent (Figure 2B). Physiologically, these approaches reduce left-sided loading by different mechanisms; clinically, the evidence supporting any of them is weaker than their physiological rationale suggests. Observational cohorts and a 2024 network meta-analysis of 45 observational studies (34,235 patients) have associated unloading with lower mortality than no unloading, but these data are vulnerable to confounding by indication, treatment-selection bias and centre-level practice, and unloading strategies may increase device-related complications (19–22); a recent two-centre target-trial emulation found no significant 60-day survival advantage with early IABP or Impella unloading; its confidence interval remained compatible with clinically relevant benefit or harm, and residual confounding limits causal inference (23). The two randomised trials of early transseptal left atrial decompression have not shown a clinical outcome benefit (24–26). EARLY-UNLOAD randomised patients on VA-ECMO regardless of loading status to routine early transseptal left atrial decompression or a conventional approach; it found no reduction in 30-day mortality and no improvement at one year in mortality or heart-failure rehospitalisation (25, 26). Because 50% of the conventional arm also received transseptal decompression, mostly as rescue a median of 21.8 h after randomisation, EARLY-UNLOAD compared early with deferred decompression rather than decompression with none. EVOLVE-ECMO, in contrast, enrolled only patients who already had signs of LV overload (significant pulmonary oedema, frothy or blood-tinged secretions, or insufficient aortic valve opening) and found that early left atrial venting did not improve its primary endpoint of VA-ECMO weaning, nor survival to discharge (24); 76.7% of its conventional arm was vented later (median 48 h vs. 2.4 h), so it too was a trial of timing; and contemporary meta-analyses of proactive vs. rescue unloading show no advantage in weaning or short-term survival (27). We therefore present the selective strategy that follows, escalation guided by objective evidence of LV distension, persistent aortic valve closure, pulmonary congestion or inadequate native ejection, as a physiology-informed default adopted in the absence of evidence rather than as an evidence-based recommendation. The optimal device, threshold and timing remain undefined; the strategy is a hypothesis that remains to be tested, and it should be abandoned if randomised data contradict it.

These approaches differ in mechanism and expected magnitude of unloading, access-site and bleeding burden, device-specific management requirements, and cost or institutional availability (Table 1). No adequately powered randomised trial has compared IABP, Impella, transseptal decompression and surgical venting head-to-head. Existing direct comparative evidence is observational: a meta-analysis comparing Impella with IABP during VA-ECMO found similar short-term mortality but more major bleeding and haemolysis with Impella (28), while a broader observational network meta-analysis suggested lower mortality with several unloading strategies vs. no unloading but also a greater complication burden (22). These findings should not be interpreted as establishing a hierarchy of clinical superiority. The broader candidacy framework that should precede device selection is shown in Figure 3.

**Figure 3:**
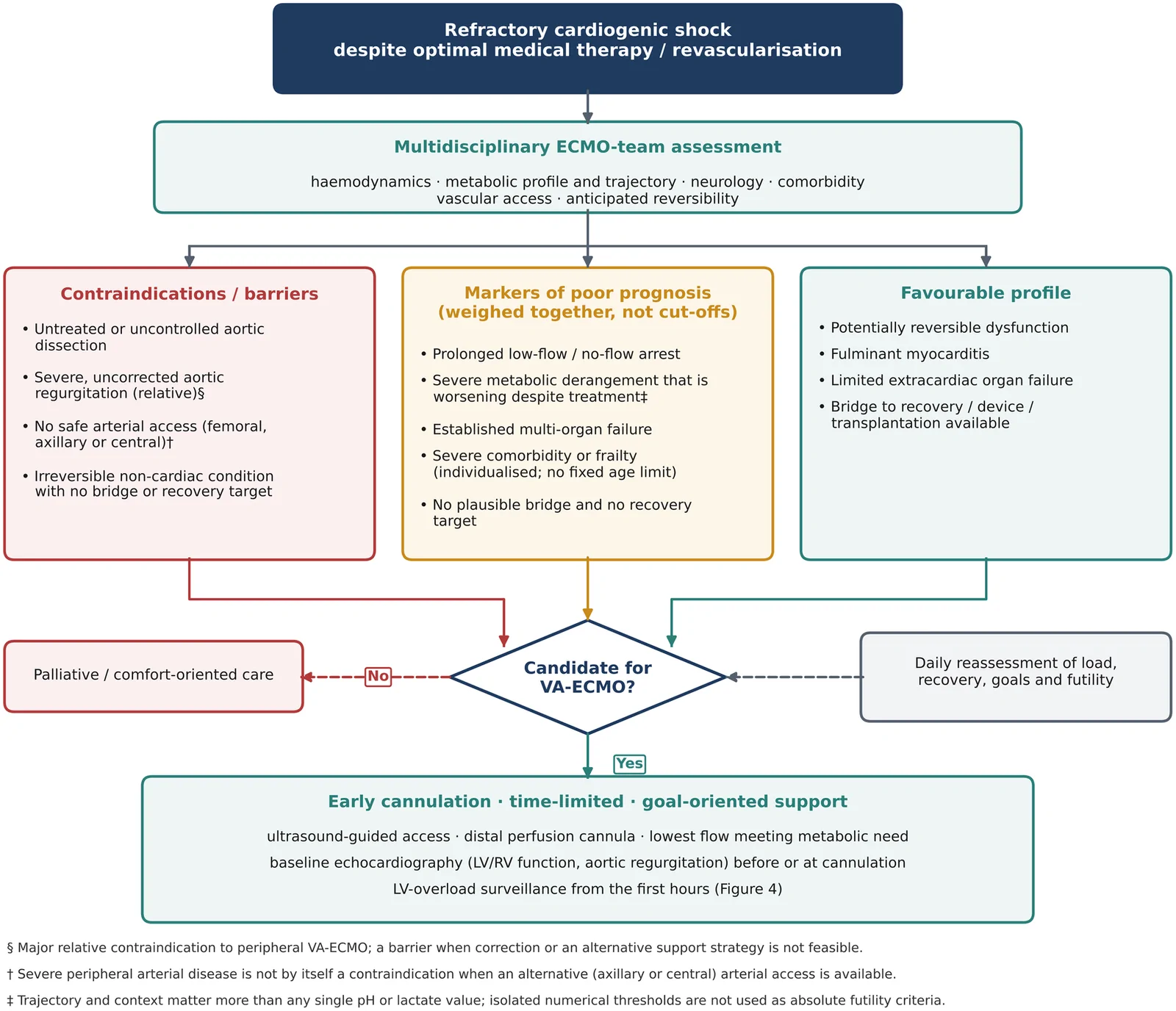
A practical framework for VA-ECMO decision-making in refractory cardiogenic shock. Within a multidisciplinary ECMO-team assessment, candidacy is weighed against contraindications and major barriers, markers of poor prognosis and features of a favourable, potentially reversible profile. Markers of poor prognosis are weighed together and by their trajectory; no single pH or lactate value and no fixed age cut-off is used as an absolute futility criterion (see Indications and patient selection). Severe peripheral arterial disease is not by itself a contraindication when an alternative (axillary or central) arterial access is available. Severe uncorrected aortic regurgitation is a major relative contraindication to peripheral VA-ECMO and becomes a barrier when correction or an alternative support strategy is not feasible. Patients who are not candidates are directed to palliative, comfort-oriented care; when support is initiated it should be time-limited and goal-oriented, delivered at the lowest flow meeting metabolic need, preceded by baseline echocardiography including assessment of aortic regurgitation, and accompanied by surveillance for ventricular overload from the first hours (Figure 4) and by daily reassessment of load, recovery, goals and futility.

## Practical algorithm for targeted LV unloading

A practical bedside approach is shown in Figure 4. The first step is to separate detection of clinically important LV overload from selection of an unloading device. First, optimise reversible determinants of loading: confirm adequate but not excessive ECMO flow, correct hypovolaemia or congestion, treat severe hypertension or vasoplegia, optimise oxygenation/ventilation and rhythm, and reassess native ejection. Second, look serially for convergent evidence of overload rather than relying on one cut-off: absent or intermittent aortic valve opening or very low pulse pressure, progressive LV dilatation, spontaneous echo contrast or intracardiac stasis/thrombus, worsening pulmonary oedema on chest radiography or lung ultrasound, persistently elevated left-sided filling pressure when invasively available, or refractory ventricular arrhythmia plausibly related to distension. Failure of these abnormalities to improve after reasonable optimisation should prompt consideration of mechanical unloading (Table 2) (14, 18, 25, 30). Table 2 specifies the instrument, proposed threshold and interpretive weight for each trigger; these thresholds are pragmatic and have not been prospectively validated. One high-risk trigger (persistent aortic valve closure with a dilating ventricle, dense spontaneous echo contrast or intracavitary thrombus, or haemorrhagic or refractory pulmonary oedema) constitutes the “emergency trigger” of Figure 4: it calls for urgent bedside reassessment to confirm the finding and, once it is confirmed and contraindications have been reviewed, for prompt consideration of a venting strategy adapted to the phenotype. An LV thrombus, for example, contraindicates a microaxial pump. Escalation is also proposed when two concordant supportive signals persist 2–6 h after optimisation. Assessment is performed within the first 6–12 h after cannulation, at least daily thereafter and after every change in pump flow, and the effect of venting is verified within 6–12 h.

**Figure 4:**
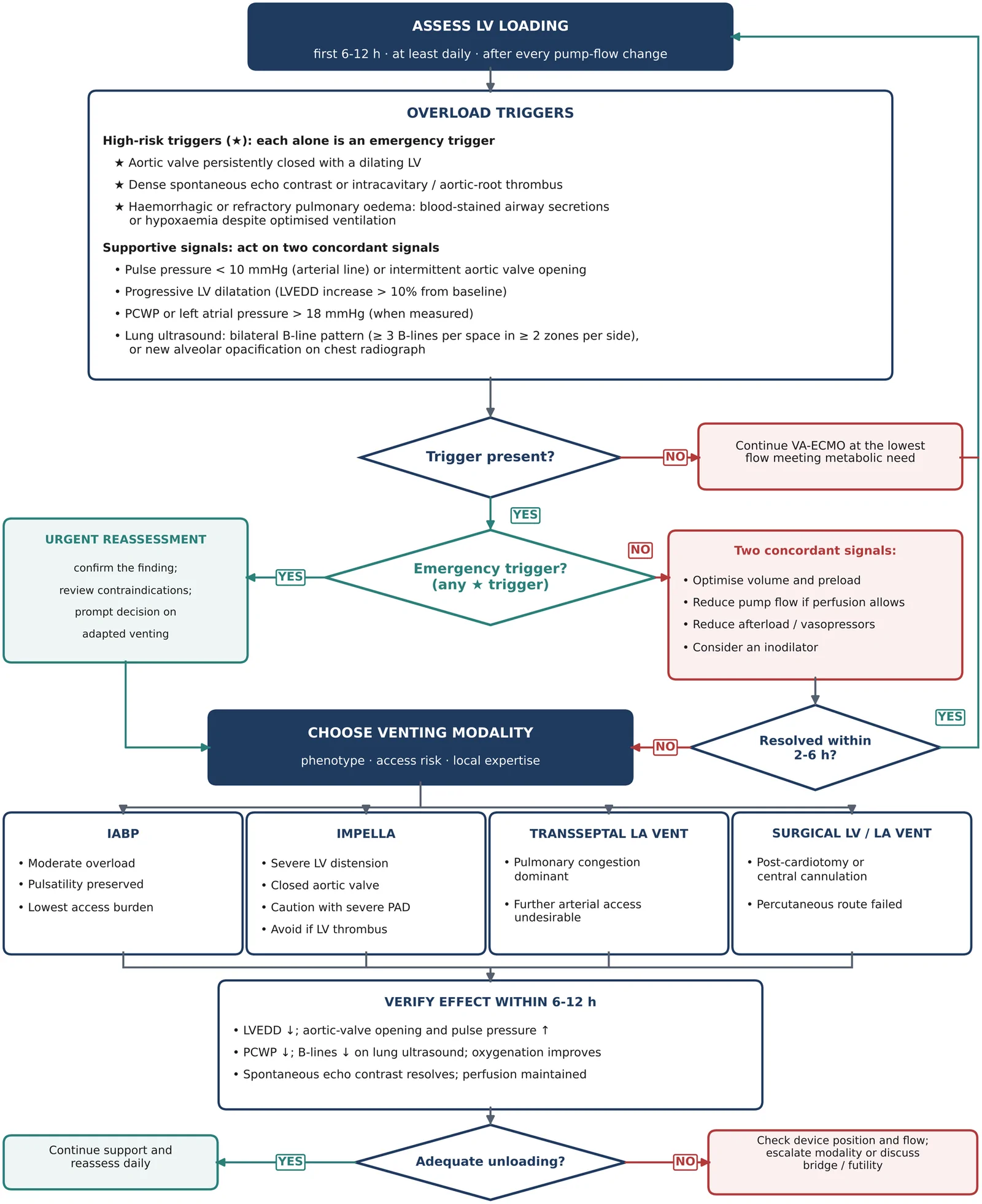
Practical step-by-step algorithm for targeted LV unloading during VA-ECMO. The algorithm separates detection of clinically important LV overload from device selection. Escalation is based on the convergence and trajectory of haemodynamic, echocardiographic and pulmonary findings after correction of reversible contributors. An emergency trigger is any one of the high-risk (★) triggers: persistent aortic valve closure with a dilating LV, dense spontaneous echo contrast or intracavitary thrombus, or haemorrhagic or refractory pulmonary oedema; it prompts urgent reassessment to confirm the finding and, after review of contraindications, prompt consideration of an adapted venting strategy (an LV thrombus, for example, contraindicates a microaxial pump). Otherwise, two concordant supportive signals (pulse pressure <10 mmHg, LV dilatation, left-sided filling pressure >18 mmHg, or a bilateral B-line pattern or alveolar opacification) lead to optimisation and reassessment within 2–6 h. Lung ultrasound B-lines are used both as a trigger and to verify the effect of venting. Thresholds are harmonised with Table 2. Device choice is matched to the required magnitude and rapidity of decompression, ventricular phenotype, anatomy, contraindications, complication profile, local expertise and device availability. Because universal prospectively validated cut-offs and randomised head-to-head device comparisons are lacking, the framework is pragmatic rather than prescriptive (18–22, 25, 28, 30). IABP, intra-aortic balloon pump; LA, left atrial; LVEDD, left ventricular end-diastolic diameter; PAD, peripheral arterial disease; PCWP, pulmonary capillary wedge pressure.

**Table 2:** Pragmatic triggers for rescue (targeted) LV unloading during VA-ECMO: instruments, thresholds, decision rule and time window.

Domain	Instrument	Findings that should raise concern (threshold)	Weight/interpretation
Aortic valve	TTE or TOE (2D and M-mode), arterial waveform	Aortic valve closed on consecutive beats with a dilating LV	★ High-risk
	TTE/TOE; arterial waveform	Absent or intermittent aortic valve opening without LV dilatation	Supportive
Haemodynamics	Arterial line (right radial preferred)	Markedly reduced pulse pressure (values <10 mmHg are commonly used as a warning sign, not a validated intervention threshold)	Supportive. Interpret in context of ECMO flow, MAP, vasoactive therapy and volume status. No single numerical value is a validated universal trigger.
	PAC (PCWP), direct LAP or LVEDP, when available	Persistently high left-sided filling pressure (>18 mmHg)	Supportive
Echocardiography	TTE/TOE; LVEDD in the same view and at the same pump flow	Progressive LV dilatation (LVEDD increase >10% from baseline); worsening LV ejection	Supportive. Trend and convergence are more important than an isolated value.
	TTE/TOE (LV cavity, aortic root)	Dense spontaneous echo contrast; intracardiac stasis or thrombus	★ High-risk
Pulmonary/clinical	Lung ultrasound	Bilateral B-line pattern: ≥3 B-lines per intercostal space in ≥2 zones per hemithorax, new or increasing	Supportive. Exclude alternative causes (pneumonia, ARDS, fluid overload from other causes).
	Chest radiograph	New or worsening pulmonary oedema: alveolar opacification	Supportive
	Airway inspection; blood gases	Blood-stained (frothy or haemorrhagic) airway secretions, or oxygenation requirement rising despite optimised ventilation	★ High-risk
	ECG monitoring	Refractory ventricular arrhythmia plausibly related to distension	Supportive. Exclude alternative causes and correct reversible loading conditions.
Response to optimisation	Repeat assessment	Persistent or worsening abnormalities despite optimisation of ECMO flow, preload/volume status, blood pressure, rhythm and gas exchange	Escalate to a mechanical unloading strategy matched to phenotype, anatomy, urgency and local expertise.
Decision rule		One high-risk (★) trigger (=emergency trigger, Figure 4), or two concordant supportive signals persisting after optimisation	★: urgent reassessment to confirm the finding, review of contraindications (e.g., LV thrombus contraindicates a microaxial pump), then prompt consideration of adapted venting. Two supportive signals: optimise, reassess within 2–6 h, vent if persisting
Time window		Assess within the first 6–12 h after cannulation, at least daily thereafter and after every change in pump flow	Verify the effect of venting within 6–12 h (Figure 4)

PAC, pulmonary artery catheter; TOE, transoesophageal echocardiography; TTE, transthoracic echocardiography. Thresholds are pragmatic and have not been prospectively validated (14, 18, 25, 30).

Device selection should then be matched to the phenotype and required magnitude and rapidity of decompression (Figure 4). IABP may be considered when modest indirect afterload reduction is sufficient and arterial anatomy is suitable; a microaxial LV pump provides direct LV unloading when more substantial or rapid ventricular decompression is required and vascular access, aortic valve anatomy and bleeding risk are acceptable; transseptal left atrial venting is an option when left atrial and pulmonary venous decompression is the principal goal; and direct surgical LV venting provides powerful decompression in post-cardiotomy or open-chest settings. These are pragmatic physiology-informed choices rather than a validated hierarchy, because randomised head-to-head evidence is lacking (18–22, 28). Neither the triggers listed in Table 2 nor the sequence shown in Figure 4 has been prospectively validated. Both represent a structured synthesis of physiological reasoning and observational data, offered to make bedside decisions explicit, reproducible and auditable rather than to prescribe them. We present them in this form deliberately: an algorithm that can be written down can also be tested, and we would regard their prospective evaluation, including the predictive value of the individual triggers, as a more tractable research question than a further trial of unloading applied to unselected patients.

## Pharmacological modulation of ventricular load

Pharmacological modulation of ventricular load may complement mechanical strategies in selected patients with adequate arterial pressure. Inodilators such as levosimendan or milrinone can increase native LV ejection while reducing systemic and pulmonary vascular resistance, whereas carefully titrated vasodilators may reduce LV afterload when hypotension and vasoplegia are absent. These physiological effects should not be equated with proven outcome benefit. In the randomised LEVOECMO trial involving 205 patients with severe, potentially reversible cardiogenic shock supported by VA-ECMO, early levosimendan did not improve successful ECMO weaning at 30 days compared with placebo (68.3% vs. 68.3%; subdistribution hazard ratio 1.02, 95% CI: 0.74–1.39) and did not reduce 60-day mortality; ventricular arrhythmias and premature treatment discontinuation were both more frequent with levosimendan, a safety signal that further argues against its routine use for ECMO weaning (31). Pharmacological measures should therefore be considered individualised adjuncts rather than substitutes for mechanical decompression when clinically important LV distension, persistent aortic valve closure or refractory hydrostatic pulmonary oedema is present.

## Right ventricular considerations

VA-ECMO's effect on the right ventricle (RV) differs from its effect on the LV: venous drainage decompresses the RV, and in predominant RV failure this decompression can itself be therapeutic. However, RV and LV function remain strongly interdependent. A distended, poorly unloaded LV raises left atrial and pulmonary venous pressures, promoting pulmonary congestion and potentially increasing pulmonary artery pressure. RV afterload becomes particularly relevant when pulmonary vascular resistance also rises, for example with hypoxaemia, hypercapnia, lung injury or reactive pulmonary vasoconstriction; conversely, a failing, dilated RV can shift the interventricular septum and impair LV filling. Dual-circulation physiology adds another layer: in peripheral VA-ECMO, antegrade native and retrograde extracorporeal flows are competitive flows that meet at the aortic mixing point. Oxygen and carbon dioxide content in the native-lung and membrane-lung zones therefore depend on native pulmonary gas exchange, pulmonary blood flow, LV ejection, extracorporeal flow and membrane-lung gas exchange (13). Serial echocardiographic assessment should therefore evaluate the RV alongside the LV, including RV size, systolic function and septal configuration, while integrating pulmonary vascular and respiratory conditions. Assessment of the right ventricle should extend beyond chamber size and qualitative systolic function to the relationship between right ventricular performance and the load it faces. Indices of right ventricular-pulmonary arterial coupling, most simply the ratio of tricuspid annular plane systolic excursion to estimated pulmonary artery systolic pressure, describe that relationship more completely than either component alone, and contemporary ELSO guidance places serial echocardiographic and extracardiac ultrasound assessment at the centre of monitoring during extracorporeal support (30). Under VA-ECMO these indices carry the same caveat as their left-sided counterparts: extracorporeal flow, intravascular volume and ventilation all modify them, so they characterise the right ventricle under a defined support condition rather than an intrinsic contractile property. Tricuspid annular plane systolic excursion has nevertheless emerged among the echocardiographic variables associated with successful separation (32). Serial biventricular assessment performed under stated flow conditions, rather than isolated left-sided measurement, is therefore the appropriate standard. Ventricular load under VA-ECMO is thus a biventricular, interdependent problem, of which LV afterload is the best-characterised but not the only component.

## Biomechanical and engineering perspective

Computational fluid dynamics (CFD), reduced-order circulation models and *in vitro* mock circulatory loops provide a mechanistic bridge between bedside observations and circuit physics. Nezami and colleagues demonstrated in patient-derived aortic models that the interaction between continuous retrograde ECMO return and pulsatile native ejection changes aortic flow distribution, pressure and the location of the mixing region as the relative contribution of native cardiac output and ECMO support changes. Subsequent computational work has extended this framework to oxygen transport and alternative cannulation configurations (14, 15).

Gasparotti and colleagues combined patient-specific CFD with an experimental mock circulatory loop and identified a flow-dependent trade-off: increasing ECMO support can restore branch-vessel perfusion while simultaneously increasing aortic pressure and LV afterload, implying that the haemodynamically optimal flow may be patient- and geometry-specific rather than simply maximal (16). Their later shape-modelling work further showed that aortic morphology itself influences the position and behaviour of the mixing region (17). These studies complement clinical trials by explaining why identical pump flows can generate different ventricular loading and regional oxygen delivery patterns across patients.

Reduced-order arterial models, including the one-dimensional pulse-wave framework developed and experimentally validated by Alastruey and colleagues, provide the mathematical basis for efficient simulation of pressure and flow propagation through compliant arterial networks and have been adapted in ECMO modelling to study mixing and cerebral oxygen delivery (33, 34). These engineering approaches remain translational rather than prescriptive: they support the ventricular-load framework and motivate patient-specific optimisation, but they do not yet define clinical intervention thresholds. These models yield a clinically testable prediction rather than a threshold. If the trade-off between branch-vessel perfusion and ventricular loading is governed in part by patient-specific arterial geometry, then the extracorporeal flow that best balances the two should differ appreciably between patients of similar body size and similar native output. That prediction is examinable at the bedside by measuring arterial pulsatility, aortic valve opening, left ventricular outflow tract velocity-time integral and regional oxygenation across a protocolised range of pump flows in the same patient. Framed this way, flow titration ceases to be a perfusion target alone and becomes a loading decision, which is precisely the reorientation this review proposes.

## Indications and patient selection

Patient selection remains central to the net benefit of VA-ECMO. Potential indications include refractory cardiogenic shock from acute coronary syndromes, fulminant myocarditis, selected refractory cardiac arrest managed with extracorporeal cardiopulmonary resuscitation (ECPR), and post-cardiotomy shock (4, 35–40). Poor neurological prognosis, prolonged low-flow states, severe metabolic derangement and established multiorgan failure are consistently associated with worse outcomes, but isolated numerical thresholds such as a single pH or lactate value should not be used as absolute futility criteria. Candidacy should be decided by a multidisciplinary ECMO team integrating shock severity, trajectory, neurological status, comorbidity, vascular access, and above all the likelihood of reversibility or a viable bridge strategy. Major anatomic or clinical barriers include uncontrolled aortic dissection, severe uncorrected aortic regurgitation (a major relative contraindication that becomes a barrier when correction or an alternative support strategy is not feasible) and situations in which safe arterial access or an acceptable bridge strategy cannot be achieved; severe peripheral arterial disease is not by itself a contraindication when an alternative axillary or central arterial access is available; advanced age and severe comorbidity require individualised assessment rather than fixed exclusion cut-offs (41). These principles are reflected in Figure 3.

## Current randomised evidence

Until recently, most evidence derived from registries and observational cohorts, with substantial selection bias and confounding by indication. Randomised trials and individual-patient-data analyses have now reshaped the field (Table 3). ECLS-SHOCK, the largest VA-ECMO trial, enrolled 417 patients with infarct-related shock and found no reduction in 30-day mortality with early routine VA-ECMO, while bleeding and vascular complications increased (5). ECMO-CS and EURO SHOCK were directionally concordant (7, 8). The neutrality of these results has proved durable rather than provisional: at one year, ECMO-CS showed no benefit of an immediate VA-ECMO strategy over early conservative management with rescue support (42). An individual-patient-data meta-analysis of VA-ECMO trials (567 patients) found no mortality benefit (odds ratio 0.93, 95% CI: 0.66–1.29), with increased major bleeding and peripheral vascular complications (6). A broader 6-month individual-patient-data analysis of active temporary MCS likewise found no significant overall mortality reduction and more major bleeding and vascular complications, although selected ST-elevation shock patients without hypoxic brain-injury risk appeared to derive benefit (9). Against this background, DanGer Shock stands apart: in 355 patients with ST-elevation myocardial infarction-related shock, a primary microaxial-flow-pump strategy reduced 180-day all-cause mortality (45.8% vs. 58.5%; hazard ratio 0.74) but increased adverse events including bleeding, limb ischaemia and renal replacement therapy (10). DanGer Shock did not compare Impella with VA-ECMO and differed in patient selection, device physiology and treatment strategy. Direct LV unloading is therefore a plausible mechanistic contributor to the divergent results, but it was not isolated by trial design and should not be presented as a proven causal explanation. Long-term follow-up of DanGer Shock, reported as a research letter, describes a durable and widening survival difference extending to ten years (absolute mortality reduction 16.3%; a mean of 600 additional days alive in the microaxial-pump group) (43). We record this finding without treating it as mechanistic support: causes of death beyond six months were not adjudicated, and a difference that widens over time is equally compatible with a systematic difference in the underlying health of the survivors in each group. It strengthens confidence that the original 180-day result was not a transient artefact; it says nothing about why the two strategies diverged.

**Table 3:** Landmark randomised trials and contemporary analyses relevant to temporary mechanical circulatory support and left-sided unloading in cardiogenic shock.

Trial/analysis (year)	Design	Population	Intervention vs. control	N	Key result
ECLS-SHOCK (2023) (5)	Randomised trial	Infarct-related shock, early revascularisation	Early routine VA-ECMO vs. medical therapy	417	No reduction in 30-day mortality; more bleeding and vascular complications
ECMO-CS (2023) (7)	Randomised trial	Severe/rapidly deteriorating shock	Immediate VA-ECMO vs. early conservative	117	No difference in composite 30-day outcome
EURO SHOCK (2023) (8)	Randomised trial	Infarct shock refractory after PCI	VA-ECMO vs. standard care	35 (17 VA-ECMO; 18 control)	Stopped early because of recruitment constraints; 30-day mortality 43.8% with VA-ECMO vs. 61.1% with standard care (HR: 0.56, 95% CI: 0.21–1.45; *p* = 0.22); bleeding and vascular complications were more frequent with VA-ECMO
IPD meta-analysis, Zeymer (2023) (6)	IPD meta-analysis	Infarct-related shock (4 RCTs)	VA-ECMO vs. medical therapy	567	OR: 0.93 (0.66–1.29) for 30-day death; bleeding OR: 2.44, vascular OR: 3.53
DanGer Shock (2024) (10)	Randomised trial	STEMI shock, LVEF <45%, no coma after OHCA	Primary microaxial flow pump (Impella CP) vs. standard care	355	180-day mortality 45.8% vs. 58.5% (HR: 0.74); more bleeding, limb ischaemia, RRT
EARLY-UNLOAD (2023) (25)	Randomised trial	Shock on VA-ECMO (LV overload not required)	Routine early transseptal left atrial decompression vs. conventional approach (rescue decompression when indicated; 50% of this arm crossed over)	116	No reduction in 30-day all-cause mortality with routine early transseptal decompression (47% vs. 45%; HR: 1.02; *p* = 0.94); pulmonary congestion resolved earlier in the early-decompression group; a comparison of timing
6-month IPD meta-analysis (2024) (9)	IPD meta-analysis	AMICS; randomised active MCS trials	Early active MCS vs. control	1,058	No significant overall 6-month mortality reduction; more major bleeding and vascular complications; selected STEMI/no hypoxic-brain-injury-risk subgroup showed benefit
EVOLVE-ECMO (2023) (24)	Randomised trial	Cardiac arrest/shock on VA-ECMO with LV overload criteria	Early transseptal LA venting vs. conventional strategy (later venting in 76.7%)	60	No improvement in VA-ECMO weaning or survival to discharge; pulmonary congestion improved earlier; a comparison of timing
LV unloading network meta-analysis (2024) (22)	Observational network meta-analysis	VA-ECMO populations; 45 observational studies	IABP, percutaneous LV assist device (pLVAD), atrial septostomy or catheter venting vs. no unloading/network comparisons	34,235	Several unloading strategies were associated with lower mortality vs. no unloading, but complication risk tended to be higher; observational evidence only
Updated unloading-timing meta-analysis (2025) (27)	Systematic review and meta-analysis	Cardiogenic shock on VA-ECMO; 8 studies	Proactive vs. passive/rescue LV unloading	2,117	No significant difference in ECMO weaning, in-hospital mortality or 30-day mortality
Target-trial emulation (2026) (23)	Target-trial emulation	Refractory cardiogenic shock on VA-ECMO; two European centres	Early IABP/Impella unloading vs. VA-ECMO alone	264	No significant 60-day survival advantage after overlap weighting; device-related complications were more frequent with unloading

EURO SHOCK was stopped early before completion of recruitment. AMICS, acute myocardial infarction-related cardiogenic shock; HR, hazard ratio; IPD, individual patient data; LV, left ventricular; LVEF, left ventricular ejection fraction; OHCA, out-of-hospital cardiac arrest; OR, odds ratio; PCI, percutaneous coronary intervention; RRT, renal replacement therapy; STEMI, ST-elevation myocardial infarction.

Two explanations compete for the divergence between these trials, and the available data do not separate them. The first is the one this review develops: the loading consequences of the support configuration differ, and a strategy that decompresses the left ventricle avoids the retrograde arterial load that peripheral VA-ECMO imposes. The second is simpler and at least as well supported: the populations were not the same. DanGer Shock excluded patients who remained comatose after out-of-hospital cardiac arrest and restricted enrolment to ST-elevation infarction with an ejection fraction below 45%, whereas the VA-ECMO trials enrolled broader populations in which patients resuscitated from cardiac arrest and patients with advanced metabolic derangement at enrolment were substantially represented. The 6-month individual-patient-data analysis of active temporary mechanical circulatory support is instructive in this respect: it was neutral overall, yet a benefit emerged in ST-elevation shock without hypoxic brain-injury risk (9). That signal is defined by patient selection rather than by device physiology, and it applies to temporary support in general rather than to microaxial unloading in particular. We therefore present ventricular load as an organising framework for bedside reasoning rather than as a demonstrated mechanism. Where the two explanations converge on the same clinical instruction, namely to support the patient in whom recovery remains plausible and to avoid imposing a load that cannot subsequently be relieved, the framework is useful irrespective of which proves correct.

This framework must also accommodate the neutral randomised trials of early left atrial decompression. EARLY-UNLOAD and EVOLVE-ECMO tested early left atrial decompression on top of VA-ECMO rather than a primary device strategy that avoids retrograde arterial return; neither demonstrated a clinical benefit from early venting (24, 25), and the 1-year EARLY-UNLOAD follow-up remained neutral (26). The two trials differ in a way that matters for the present hypothesis. EARLY-UNLOAD enrolled patients regardless of loading status and is therefore a test of routine early decompression; it does not address documented overload. EVOLVE-ECMO enrolled only patients with documented overload and is therefore the more direct test of the selective strategy proposed here. Its neutral result does not support the hypothesis that earlier venting of patients with documented overload improves weaning or survival, although the small trial cannot exclude a clinically important effect. It does not, however, test venting against no venting: about half of the EARLY-UNLOAD conventional arm and three-quarters of the EVOLVE-ECMO conventional arm were decompressed later, when overload persisted or progressed, and both reported earlier improvement in pulmonary-congestion measures with early venting (24–26). Read together, the trials suggest that, once overload is recognised, deferring decompression until it proves persistent despite optimisation did not lead to worse outcomes than immediate decompression, which is consistent with the stepwise rule in Table 2 and Figure 4, while leaving untested whether decompression itself, as opposed to its timing, improves outcome. Both trials were small and powered for short-term endpoints, so a modest benefit or harm cannot be excluded. Contemporary meta-analyses of proactive vs. rescue/passive unloading have likewise not demonstrated a clear improvement in ECMO weaning or short-term mortality (27). At the same time, observational cohorts and network meta-analyses continue to report associations between unloading and improved survival (19–22), whereas a recent target-trial emulation found no 60-day survival advantage with early unloading (23). The discordance is best interpreted as persistent uncertainty rather than proof for or against unloading. It supports a more specific hypothesis: the haemodynamic consequences of a support configuration matter, but the benefit of adding an unloading intervention probably depends on whether clinically important LV overload is actually present, on the unloading method and magnitude, on timing, and on competing procedural risks. Ongoing randomised trials and mechanistic studies are needed to define which patients, if any, benefit from pre-emptive rather than rescue unloading.

## Controversies and unresolved questions

### Which patients, and when?

The randomised data do not abolish a role for VA-ECMO; they narrow it. The trials primarily evaluated early routine support in infarct-related shock, and some were limited by factors such as delayed initiation, advanced metabolic derangement at enrolment or crossover to rescue support in control groups. The unresolved clinical question is the therapeutic window: support must be initiated early enough to prevent irreversible organ injury, while avoiding device-related harm in patients likely to recover with conventional therapy and avoiding futile support once irreversible multiorgan failure is established.

### Device selection and LV unloading

DanGer Shock reframes the discussion from “ECMO yes/no” to “which support, for which physiology?” A microaxial pump directly unloads the LV, whereas peripheral VA-ECMO can increase effective LV afterload; the two are complementary rather than interchangeable. Whether combining them (ECMELLA) improves outcomes, and whether unloading should be routine or reserved for documented overload, remains under prospective study. Current guidance remains selective rather than supportive of routine device use. The 2023 ESC acute coronary syndrome guideline states that the clinical benefit of percutaneous MCS and/or VA-ECMO in acute coronary syndrome complicated by cardiogenic shock remains unclear, while MCS may be considered in selected patients with severe or refractory shock (44). The 2025 ACC/AHA/ACEP/NAEMSP/SCAI acute coronary syndrome guideline considers a microaxial intravascular flow pump reasonable in selected patients with STEMI and severe or refractory cardiogenic shock (Class 2a), while routine IABP or VA-ECMO in AMI-related cardiogenic shock is not recommended because of lack of survival benefit (Class 3: No Benefit) (45). VA-ECMO should therefore be viewed as a time-limited, goal-oriented therapy with explicit, repeated reassessment of indication, recovery, bridge options and futility.

### Implementation and management

Cannulation is generally performed percutaneously under ultrasound guidance or by surgical cut-down (46), with venous drainage and arterial return configured according to anatomy and clinical context; a distal perfusion cannula is commonly used with femoral arterial cannulation to reduce limb-ischaemia risk. Circuit flow should be titrated to restore adequate systemic perfusion while avoiding unnecessary ventricular loading. Beyond the ventricular-load surveillance detailed in this review, routine ECMO care requires systematic assessment of end-organ function, oxygen delivery, haemostasis, circuit integrity and vascular complications. Anticoagulation should be individualised according to bleeding and thrombotic risk and institutional/ELSO-based protocols. These broader aspects of ECMO management are intentionally not reviewed comprehensively here and should be guided by dedicated ELSO and society recommendations (41).

### Complications

Complications are frequent and materially affect the risk-benefit balance, an issue underscored by the excess bleeding and vascular events seen even in the randomised trials (Table 4). Their anticipation and early management are as important to outcome as the decision to cannulate.

**Table 4:** Principal complications of peripheral VA-ECMO.

Complication	Frequency	Comment
Haemorrhage	Variable across cohorts (∼25%–40%)	Cannulation-site, gastrointestinal, thoracic or intracranial; associated with anticoagulation, coagulopathy and acquired von Willebrand syndrome (12, 47, 48)
Limb ischaemia	Variable across cohorts (∼10%–20%)	Ipsilateral to arterial cannulation; a distal perfusion cannula reduces risk (12, 49)
Thromboembolism/circuit thrombosis	Variable; depends on definitions and populations	Deep-vein thrombosis, pulmonary embolism and stroke; circuit thrombosis is a life-threatening emergency requiring urgent circuit assessment and, when indicated, exchange (12, 47, 48)
Differential oxygenation with regional hypoxaemia	Configuration-dependent	When native-lung gas exchange is impaired, native LV ejection may supply a hypoxaemic native-lung zone proximal to the mixing point while the membrane-lung zone remains well oxygenated. Management requires right-upper-extremity arterial monitoring and correction of the underlying gas exchange and support imbalance; circuit reconfiguration may be required in selected cases (13, 50).
Infection	∼20%	Cannula-site and bloodstream infections; risk rises with support duration (51)

Frequencies are drawn from adult cohorts, reviews and meta-analyses (12, 47, 48).

### Weaning and outcomes

Weaning is considered once recovery appears sufficient and should be assessed during a controlled reduction in ECMO support rather than by a single static threshold. A structured stepwise low-flow trial for assessing separation readiness is summarised in Table 5. In small observational series, LVEF >20%–25% and lateral mitral annular systolic (S′) velocity >∼6 cm/s have been associated with successful separation, but these values are load- and protocol-dependent and should be integrated with aortic valve opening, arterial pulsatility, LVOT velocity-time integral, biventricular function and invasive haemodynamics when available (52–56). Validated scores such as SAVE and ENCOURAGE, together with shock staging systems, can support population-level risk stratification and communication but have limited precision for individual futility decisions and should not override serial clinical assessment (57–60).

**Table 5:** Operational low-flow trial for assessment of VA-ECMO separation readiness.

Step	Bedside action	Acceptable findings/targets
1. Preconditions	Precipitating cause treated (e.g., revascularisation completed, arrhythmia controlled, myocarditis or other reversible insult past its acute phase); confirm stable rhythm, gas exchange and perfusion; optimise preload/afterload; exclude active LV overload	MAP ≥65 mmHg on stable or decreasing low-dose vasoactive support; no escalating vasopressors in the preceding 12–24 h; lactate <2 mmol/L or falling; no worsening pulmonary oedema; acceptable native-lung gas exchange (right radial SpO_2_ ≥ 92% with ventilator FiO_2_ ≤ 0.5)
2. Stepwise reduction	Reduce ECMO flow in protocolised steps; ∼0.5–1.0 L/min (or 10%–20%) decrements are commonly used examples, down to the lowest flow allowed by the circuit and anticoagulation strategy (typically 1.0–1.5 L/min). Reassess after several minutes and use smaller decrements when needed to assess AV opening/distension	No hypotension, major tachycardia, desaturation or recurrent congestion
3. Echo + haemodynamics	At each step assess AV opening, pulse pressure, LVOT-VTI, lateral mitral S′, LV size/function, RV size/function, MR, pulmonary congestion (lung ultrasound); record filling pressures if a PAC or LA line is present	Regular AV opening; sustained pulsatility; LVOT-VTI commonly ≥10–12 cm; LVEF commonly ∼20%–25%; lateral S′ ≥ 6 cm/s; left-sided filling pressure (PCWP or LAP) < 18 mmHg and not rising; RV not dilating (RV/LV end-diastolic area ratio <1, no new septal flattening) with CVP <12 mmHg and not rising; RV stable/improving. Values are supportive, not absolute
4. Perfusion	Reassess lactate, urine output, skin perfusion and ScvO_2_/SvO_2_ if available	No evidence of deteriorating systemic oxygen delivery: SvO_2_ or ScvO_2_ ≥ 65% (interpreted according to sampling site) without a fall of >10 percentage points from baseline; lactate not rising; urine output ≥0.5 mL/kg/h
5. Decision	If all domains remain stable at minimal support, consider brief trial-off/clamping according to local protocol and multidisciplinary decannulation planning	Abort and return to the last tolerated flow if instability, recurrent LV overload or RV failure develops. Operational abort criteria: MAP <65 mmHg or a fall of >10 mmHg from baseline (64); need to escalate vasoactive support; loss of AV opening or LVOT-VTI <10 cm; LV dilatation or PCWP/LAP ≥18 mmHg; RV dilatation (RV/LV ≥1) with CVP rising above the <12 mmHg target (65), judged clinically; SvO_2_/ScvO_2_ < 60% or a fall of >10 percentage points; SpO_2_ < 90%; new sustained arrhythmia

CVP, central venous pressure; LAP, left atrial pressure; PAC, pulmonary artery catheter; SpO_2_, peripheral oxygen saturation. Targets are pragmatic, derived from observational series and expert protocols, and have not been prospectively validated (41, 52–56, 64, 65).

## Reading the loaded ventricle: assessing recovery and futility on VA-ECMO

Serial assessment of ventricular loading and myocardial recovery is central to time-limited VA-ECMO support. Clinicians must determine whether recovery is sufficient to permit reduction or withdrawal of support and, conversely, whether continued support remains biologically and therapeutically plausible. The following section summarises what currently available tools can and cannot establish and highlights areas in which advanced imaging remains investigational.

### Haemodynamic and biochemical signals

Haemodynamic assessment provides an indirect but clinically useful view of ventricular loading and recovery. Recovery of arterial pulsatility, aortic valve opening and increasing LVOT velocity-time integral during comparable support conditions are more direct bedside indicators of increasing native LV ejection. By contrast, lactate clearance, falling vasoactive requirements and improving venous oxygen saturation indices primarily reflect the adequacy of global oxygen delivery and tissue perfusion and may improve because of extracorporeal support even without myocardial recovery. Central, pulmonary artery or pre-oxygenator venous oxygen saturation should therefore be interpreted according to sampling site, drainage configuration, extracorporeal flow and the presence of dual circulation rather than as interchangeable measures of native cardiac output (13). Persistent loss of pulsatility, absent aortic valve opening or worsening tissue perfusion markers despite apparently adequate circuit support should prompt reassessment. When invasive monitoring is clinically justified, pulmonary artery catheter-derived pulmonary capillary wedge pressure (PCWP), direct left atrial pressure (LAP) or LV end-diastolic pressure (LVEDP) can complement echocardiography by identifying elevated left-sided filling pressures and by characterising right-sided pressures and pulmonary vascular load. Lung ultrasound provides a repeatable bedside assessment of pulmonary congestion and complements cardiac imaging; contemporary ELSO guidance places echocardiography and extracardiac ultrasound at the centre of assessment of ventricular loading conditions and pulmonary pathology throughout ECMO support (30). Serial troponin and natriuretic peptide kinetics may provide complementary information on myocardial injury and wall stress, but neither is sufficiently specific or validated to guide unloading or weaning in isolation. These measurements are best interpreted as a multimodal trajectory under known circuit flow conditions rather than as standalone thresholds.

### Echocardiography: the standard, and its ceiling

Serial transthoracic or transoesophageal echocardiography remains the principal bedside imaging tool: it tracks ventricular size and function, detects the distending or non-ejecting LV, screens for intracardiac stasis or thrombus, evaluates the RV and valve opening, and supports weaning assessment during controlled reductions in ECMO flow (30, 32, 52–54). Commonly cited low-flow features associated with successful separation include an LV ejection fraction above roughly 20%–25%, lateral mitral annular systolic (S′) velocity above approximately 6 cm/s and an adequate LVOT velocity-time integral (52–54). These values should not be treated as fixed cut-offs: every echocardiographic index is influenced by preload, afterload, vasoactive therapy and circuit flow. A single measurement therefore describes the ventricle under a particular loading condition rather than intrinsic contractility alone. Consistent with this, a systematic review and meta-analysis of weaning predictors concluded that assessment of separation readiness should integrate multiple haemodynamic, biochemical and echocardiographic variables, including pulse pressure, LVEF and LVOT velocity-time integral, rather than relying on any single predictor (56).

Deformation imaging, including global longitudinal strain and myocardial work indices that incorporate estimated afterload, may provide incremental information beyond conventional ejection fraction and has been explored in patients supported with VA-ECMO (61). Its load dependence has been quantified directly. In 46 patients on peripheral VA-ECMO (45 assessed in the acute phase and 36 in the delayed phase) studied at 50%, 100% and 120% of target pump flow, Ng and colleagues showed that LV global longitudinal strain, ejection fraction and LVOT velocity-time integral all worsened as extracorporeal flow increased, a change that alters loading conditions by raising afterload while also reducing venous return to the left heart; this afterload sensitivity was much greater in the acute phase (around day 1) than a few days later (relative change in strain about 73% vs. 23%), so the same ventricle can appear markedly different depending on the flow at which it is imaged and on the time since cannulation (62). Pressure-volume modelling of the Starling relationship under VA-ECMO provides the mechanistic explanation: in a poorly contractile, afterload-sensitive left ventricle, the rise in systemic pressure produced by extracorporeal flow shifts the operating point towards higher end-diastolic volume and filling pressure, so that LV distension and a rising wedge pressure can occur without any increase in venous return, whereas the same rise in pressure has little effect on a ventricle with preserved contractility (63). However, strain remains load-sensitive, image quality can be challenging, and acquisition/analysis protocols are not yet standardised for ECMO. These techniques should therefore be described as promising tools for serial phenotyping rather than as validated measures of load-independent contractility, and any strain or ejection-phase index should be reported together with the pump flow and arterial pressure at which it was acquired. Prospective studies are needed to determine whether deformation imaging improves prediction of myocardial recovery or successful separation beyond conventional echocardiography and haemodynamics.

### Operational low-flow trial before VA-ECMO separation

Interpretation of a failed low-flow trial. A failed trial indicates that the ventricle cannot yet sustain the circulation under the conditions of that trial; it does not by itself establish absence of recovery. Its interpretation depends on why and how it failed. Failure driven by a correctable factor (hypovolaemia or excessive preload, vasoplegia, arrhythmia, residual ischaemia, hypoxaemia, hypercapnia or a raised pulmonary vascular resistance) should lead to correction and a repeat trial, usually after 24–48 h (64, 65). The pattern of failure is informative: failure with a dilating LV, loss of aortic valve opening or rising left-sided filling pressure points to insufficient LV reserve and may justify LV unloading or transition to a configuration that unloads the LV, whereas failure with RV dilatation and rising CVP points to RV dysfunction or increased RV afterload and may call for RV-directed therapy or support. Repeated failure over several days without improvement in the trajectory of native ejection should trigger an explicit discussion of the bridge strategy, namely durable LV assist device, transplantation or palliation (Table 6), rather than indefinite repetition of trials.

**Table 6:** A bedside decision loop for VA-ECMO.

Stage	Assess and decide
Before cannulation	Reversibility of the cardiac insult; lactate; extracardiac organ failure; neurological status; baseline transthoracic (or transoesophageal) echocardiography of LV/RV size and function and aortic regurgitation severity. Severe uncorrected aortic regurgitation is a major relative contraindication to peripheral VA-ECMO because retrograde flow can worsen LV distension; it becomes a barrier when correction or an alternative support strategy is not feasible. Assess the aorta and arterial access.
First 6–12 h	Is perfusion restored? Is lactate clearing? Is pulse pressure returning and does the aortic valve open? This is also the window of highest risk for acute LV overload in a severely failing ventricle: assess early for a dilating, poorly ejecting LV, spontaneous echo contrast, persistent aortic valve closure or worsening pulmonary oedema, and be prepared to escalate to unloading sooner than the routine daily reassessment below if distension is already evident.
Daily	Reassess biventricular loading, aortic valve opening, pulmonary congestion, native output, complications and the continued validity of the bridge strategy
Low-flow (weaning) trial	Assess whether recovery remains adequate during a standardised reduction in ECMO support, integrating echocardiography, arterial pulsatility and haemodynamics
No recovery/no bridge	Explicit futility discussion; transition to a durable device, transplantation or palliation

### Beyond echocardiography: an unexplored frontier

Outside the intensive care unit, myocardial viability, perfusion and tissue characteristics can be evaluated using dobutamine or deformation stress echocardiography, cardiac magnetic resonance with late gadolinium enhancement and parametric mapping, and nuclear techniques such as FDG-PET and perfusion imaging. A substantial body of evidence links viable, hibernating myocardium to functional recovery after revascularisation and to prognosis (66). Almost none of these approaches has been validated during VA-ECMO. Important barriers include the hazards of transporting an unstable, cannulated patient, device-related constraints on magnetic resonance, altered perfusion and tracer kinetics during retrograde ECMO flow, and the fact that the acutely stunned or oedematous myocardium of cardiogenic shock differs from the chronic hibernating myocardium in which conventional viability thresholds were established.

These limitations concern feasibility, device compatibility and external validity of existing viability thresholds. They do not establish that advanced imaging cannot contribute to assessment during mechanical support, but current evidence is insufficient for clinical implementation. Future studies should evaluate point-of-care or ECMO-compatible imaging, acquisition during controlled low-flow weaning conditions, and imaging or biomarker approaches capable of distinguishing reversible stunning from irreversible myocardial injury.

## From assessment to decision

The overall decision loop, from pre-cannulation assessment to daily reassessment, the low-flow weaning trial and a formal futility discussion, is summarised in Table 6.

## Limitations

The selection of evidence in this review reflects the authors' judgement of relevance rather than a prespecified protocol, and no formal risk-of-bias assessment was undertaken. The central proposition, that peripheral VA-ECMO is usefully managed as a problem of ventricular load, is an interpretive framework derived from trials that were not designed to test it, and it is not established as a causal mechanism. The practical instruments that follow from it, in particular the triggers for rescue unloading and the operational low-flow protocol, are expert syntheses rather than validated tools, and the reference values they contain are drawn from observational series in which loading conditions were rarely reported. We have deliberately restricted the treatment of routine extracorporeal care (anticoagulation, circuit surveillance, end-organ monitoring, transfusion and infection control) to what the loading argument requires, and readers should consult dedicated society and ELSO documents for those domains (41). Finally, the review is weighted towards the left ventricle: right ventricular loading and biventricular interaction are addressed but remain less well characterised under extracorporeal support, and the imaging approaches we identify as promising are at present research tools rather than clinical instruments.

## Perspectives

The immediate priority is better patient selection and physiological phenotyping: identifying, before irreversible organ injury, patients in whom temporary support offers a credible bridge to recovery or definitive therapy, and distinguishing those who develop clinically important LV overload from those who do not. Randomised and mechanistic studies should clarify whether unloading benefits selected phenotypes, which modality produces the most appropriate degree of decompression, and how timing changes the balance between haemodynamic benefit and procedural harm (67).

Beyond clinical trials, mechanistic and engineering studies, including mock circulatory loops, pressure-volume-loop modelling and animal comparisons of IABP, microaxial pumps, septostomy and surgical venting under matched loading conditions, could quantify the magnitude of unloading achieved by each modality independently of patient selection and treatment timing. Such studies could complement the qualitative comparison in Table 1 and help explain differences in clinical effect.

Circuit miniaturisation and improved pumps facilitate transfer and earlier mobilisation, and alternative access sites (axillary, carotid, central) extend applicability (68). Structured, high-volume ECMO programmes and protocolised care remain associated with better outcomes (68, 69). Finally, prognostic modelling, including machine-learning approaches to anticipate complications and support futility decisions, may refine bedside judgement, but requires prospective validation before adoption.

## Conclusions

VA-ECMO can provide profound circulatory support, but its value depends on disciplined use in appropriately selected patients and on active management of the loading conditions created by the circuit. Contemporary randomised evidence shows that routine early VA-ECMO in infarct-related shock does not improve survival and increases complications, while DanGer Shock demonstrates that a different support strategy can improve outcomes in a narrowly selected population. That contrast supports, but does not prove, the importance of ventricular loading as a mechanistic framework; differences in patient selection between these trials provide a competing explanation that the available data cannot exclude. Peripheral VA-ECMO should therefore be managed by integrating systemic perfusion with serial assessment of LV and RV load, aortic valve opening, pulmonary congestion and myocardial recovery, and by reserving invasive unloading for a clearly defined physiological objective rather than applying it reflexively. The major unresolved challenge is how best to distinguish reversible stunning from irreversible myocardial injury under changing support conditions. Today that judgement relies on serial haemodynamics and echocardiography; future work should determine whether deformation imaging, advanced perfusion/viability imaging and other ECMO-compatible measures can add clinically useful information. The goal is not simply to deliver flow, but to use temporary support in a way that preserves the possibility of recovery while recognising failure of the bridge early enough to change strategy.

## Glossary

ARDS: acute respiratory distress syndrome
AV: aortic valve
CI: confidence interval
CFD: computational fluid dynamics
CVP: central venous pressure
ECG: electrocardiogram
ECMELLA: VA-ECMO combined with a microaxial pump (Impella)
ECMO: extracorporeal membrane oxygenation
ECPR: extracorporeal cardiopulmonary resuscitation
ELSO: Extracorporeal Life Support Organization
ESC: European Society of Cardiology
FDG-PET: fluorodeoxyglucose positron-emission tomography
FiO_2_: fraction of inspired oxygen
HR: hazard ratio
IABP: intra-aortic balloon pump
IPD: individual patient data
LA: left atrium
LAP: left atrial pressure
LV: left ventricle
LVEDD: left ventricular end-diastolic diameter
LVEDP: left ventricular end-diastolic pressure
LVEF: left ventricular ejection fraction
LVOT: left ventricular outflow tract
MAP: mean arterial pressure
MCS: mechanical circulatory support
MR: mitral regurgitation
OHCA: out-of-hospital cardiac arrest
OR: odds ratio
PAC: pulmonary artery catheter
PAD: peripheral arterial disease
PCI: percutaneous coronary intervention
PCWP: pulmonary capillary wedge pressure
pLVAD: percutaneous left ventricular assist device
RRT: renal replacement therapy
RV: right ventricle
SCAI: Society for Cardiovascular Angiography and Interventions
ScvO_2_: central venous oxygen saturation
SpO_2_: peripheral oxygen saturation
STEMI: ST-elevation myocardial infarction
SvO_2_: mixed venous oxygen saturation
TAPSE: tricuspid annular plane systolic excursion
TOE: transoesophageal echocardiography
TTE: transthoracic echocardiography
VA-ECMO: veno-arterial extracorporeal membrane oxygenation
VTI: velocity-time integral.
